# The epidemiology of residual *Plasmodium falciparum* malaria transmission and infection burden in an African city with high coverage of multiple vector control measures

**DOI:** 10.1186/s12936-016-1340-4

**Published:** 2016-05-23

**Authors:** Daniel Msellemu, Hagai I. Namango, Victoria M. Mwakalinga, Alex J. Ntamatungiro, Yeromin Mlacha, Zacharia J. Mtema, Samson Kiware, Neil F. Lobo, Silas Majambere, Stefan Dongus, Christopher J. Drakeley, Nicodem J. Govella, Prosper P. Chaki, Gerry F. Killeen

**Affiliations:** Environmental Health and Ecological Sciences Thematic Group, Ifakara Health Institute, Dar es Salaam, United Republic of Tanzania; Department of Immunology and Infection, London School of Hygiene and Tropical Medicine, London, UK; School of Biological Sciences, University of Nairobi, Nairobi, Kenya; School of Public Health, Faculty of Health Sciences, University of the Witwatersrand, Johannesburg, South Africa; Department of Vector Biology, Liverpool School of Tropical Medicine, Liverpool, UK; Department of Mathematics, Statistics and Computer Science, Marquette University, Milwaukee, WI USA; Eck Institute for Global Health, Notre Dame University, Notre Dame, IN USA; Department of Epidemiology and Public Health, Swiss Tropical and Public Health Institute, Basel, Switzerland; University of Basel, Basel, Switzerland

**Keywords:** Malaria, *Plasmodium*, Mosquito, *Anopheles*, Vector control, Larval source management, Housing, Window screening, Chronic infection, Long-lasting insecticidal net

## Abstract

**Background:**

In the Tanzanian city of Dar es Salaam, high coverage of long-lasting insecticidal nets (LLINs), larvicide application (LA) and mosquito-proofed housing, was complemented with improved access to artemisinin-based combination therapy and rapid diagnostic tests by the end of 2012.

**Methods:**

Three rounds of city-wide, cluster-sampled cross-sectional surveys of malaria parasite infection status, spanning 2010 to 2012, were complemented by two series of high-resolution, longitudinal surveys of vector density.

**Results:**

Larvicide application using a granule formulation of *Bacillus thuringiensis* var. *israelensis* (*Bti*) had no effect upon either vector density (P = 0.820) or infection prevalence (P = 0.325) when managed by a private-sector contractor. Infection prevalence rebounded back to 13.8 % in 2010, compared with <2 % at the end of a previous *Bti* LA evaluation in 2008. Following transition to management by the Ministry of Health and Social Welfare (MoHSW), LA consistently reduced vector densities, first using the same *Bti* granule in early 2011 [odds ratio (OR) (95 % confidence interval (CI)) = 0.31 (0.14, 0.71), P = 0.0053] and then a pre-diluted aqueous suspension formulation from mid 2011 onwards [OR (95 % CI) = 0.15 (0.07, 0.30), P ≪ 0.000001]. While LA by MoHSW with the granule formulation was associated with reduced infection prevalence [OR (95 % CI) = 0.26 (0.12, 0.56), P = 0.00040], subsequent liquid suspension use, following a mass distribution to achieve universal coverage of LLINs that reduced vector density [OR (95 % CI) = 0.72 (0.51, 1.01), P = 0.057] and prevalence [OR (95 % CI) = 0.80 (0.69, 0.91), P = 0.0013], was not associated with further prevalence reduction (P = 0.836). Sleeping inside houses with complete window screens only reduced infection risk [OR (95 % CI) = 0.71 (0.62, 0.82), P = 0.0000036] if the evenings and mornings were also spent indoors. Furthermore, infection risk was only associated with local vector density [OR (95 % CI) = 6.99 (1.12, 43.7) at one vector mosquito per trap per night, P = 0.037] among the minority (14 %) of households lacking screening. Despite attenuation of malaria transmission and immunity, 88 % of infected residents experienced no recent fever, only 0.4 % of these afebrile cases had been treated for malaria, and prevalence remained high (9.9 %) at the end of the study.

**Conclusions:**

While existing vector control interventions have dramatically attenuated malaria transmission in Dar es Salaam, further scale-up and additional measures to protect against mosquito bites outdoors are desirable. Accelerated elimination of chronic human infections persisting at high prevalence will require active, population-wide campaigns with curative drugs.

**Electronic supplementary material:**

The online version of this article (doi:10.1186/s12936-016-1340-4) contains supplementary material, which is available to authorized users.

## Background

Cities represent perhaps the best opportunities to eliminate local malaria transmission for a number of reasons [1–3]. First, dense aggregation of humans in urban areas reduces malaria transmission intensity, by simply diluting the biting burden created by local vector populations across larger numbers of people [4, 5]. Second, while a certain amount of human activity can increase the availability of aquatic habitat for vectors, the planning, construction and drainage processes associated with urban development can dramatically reduce it [6]. Third, cities often have far better infrastructure, health services, access to goods, institutional capacity and governance than rural areas, so some supplementary interventions, such as intensive larval source management (LSM), may be more feasible and effective than in rural areas [1–3, 7, 8].

Dar es Salaam in the United Republic of Tanzania is a typical African coastal city with ideal climatic conditions for malaria transmission, where *Plasmodium falciparum* is transmitted both indoors and outdoors [9, 10] by some of the most efficient vectors in the world, specifically *Anopheles gambiae*, *Anopheles arabiensis* and *Anopheles funestus* [11]. While Dar es Salaam historically experienced intense transmission and high infection prevalence, an operational research programme to develop and evaluate new municipal systems for larvicide application (LA) [12, 13] coincided with spontaneous uptake of window screening [14] to prevent mosquito entry, resulting in an overall decline of infection prevalence from 21 % in 2004 to only 2 % by 2008 [15].

Since then, considerable progress has recently been made in Dar es Salaam to not only ensure high coverage of standard interventions like rapid diagnostic tests (RDTs), artemisinin-based combination therapy (ACT) and long-lasting insecticidal nets (LLINs), but also supplementary vector control measures, specifically mosquito-proofed housing and regular LA with microbial active ingredients [9, 13–16]. This observational study in Dar es Salaam evaluates the influence of all these interventions upon malaria vector density and infection burden, and assesses opportunities for further progress towards elimination of malaria transmission from the city.

## Methods

### Study area

**Fig. 1:**
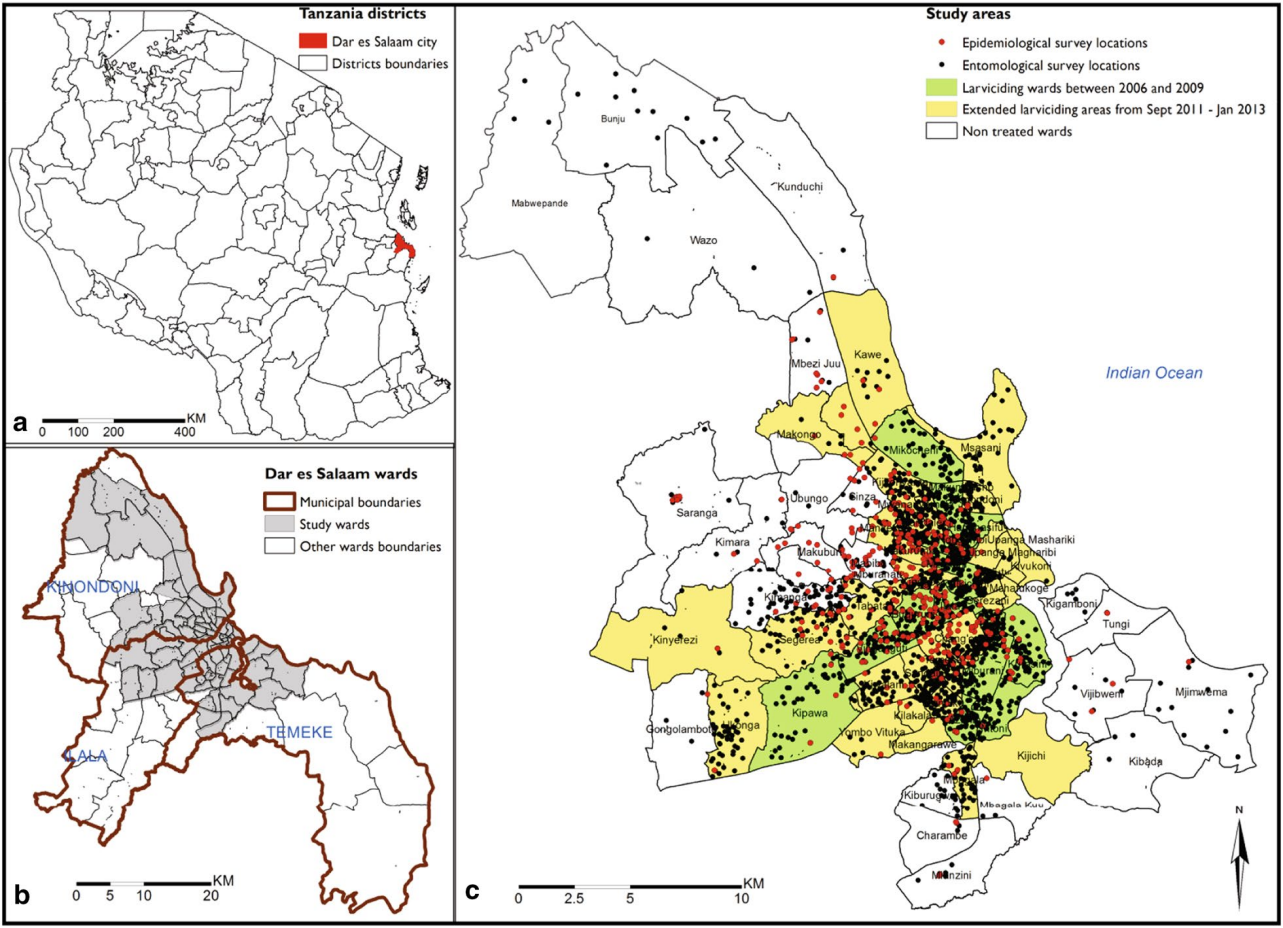
Map of where Dar es Salaam city region is located within Tanzania (**a**), the study area within the city and its three municipalities (**b**), and the survey locations and wards in which larvicides were applied (**c**)

Dar es Salaam is the biggest and most economically important city in the United Republic of Tanzania, situated on the shores of the Indian Ocean (Fig. 1). The city has 4.36 million inhabitants [17] and is among the world’s ten fastest growing cities [18], with an annual growth rate of 5.6 % [17]. However this growth is unguided, resulting in about 70 % of its inhabitants living in informal settlements [19]. Challenges resulting from unguided growth are huge and have exposed the city to floods, scarcity of piped water, overcrowding, poor sanitation, poor waste management, inadequate housing, unplanned settlement, and insecurity of residence tenure [20], all of which increase malaria transmission hazard while also increasing the vulnerability of residents to transmission exposure [21, 22].

Administratively, the city comprises three municipalities namely Ilala, Kinondoni and Temeke, which are collectively divided into 90 wards [17] (Fig. 1). The wards are further divided into smaller neighbourhood units called mitaa (a *Kiswahili* word for street, written in the singular form as *mtaa*) [23]. Each *mtaa* is subdivided into ten-cell units (TCUs), comprising clusters of approximately 10–20 houses, although some TCUs may contain more than 100 houses [23]. TCUs were the main units of sampling and analysis in this study, and also represent the geographic units that were mapped and allocated to individual community-based staff for implementation of LA [12, 23, 24]. The study area consists of the 71 urban and semi-urban wards (Fig. 1) which have been mapped in detail down to the level of TCU boundaries [23, 24], encompassing an overall population of 3.6 million living in >12,000 TCUs with >543,000 enumerated households.

Dar es Salaam has a well-documented history of successful health sector reform and malaria control programmes, with a particularly strong record of successful vector control operations [25–36]. Important decentralization, management and financing reforms of the health system in Dar es Salaam in the 1990s [26] not only improved the clinical services provided by the Ministry of Health and Social Welfare (MoHSW) [37], they also subsequently provided a suitable administrative platform for vertical but decentralized and community-based implementation of preventative public health measures, including vector control [12, 13, 38].

### Intervention scenario

**Fig. 2:**
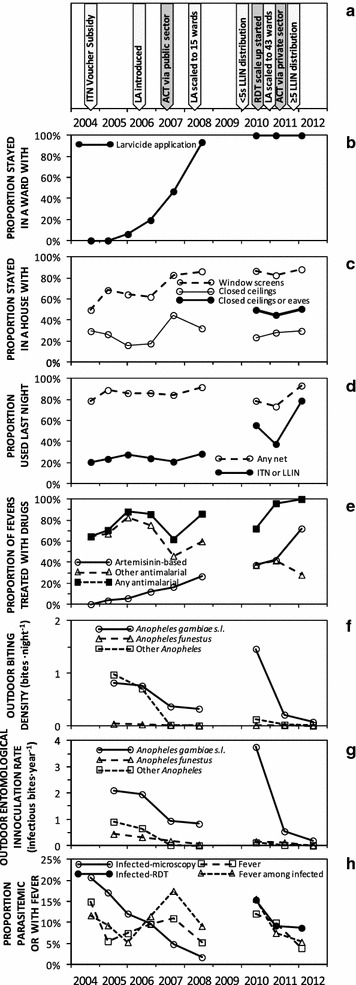
Long-term trends in coverage with malaria control interventions, entomological malaria transmission hazard, and prevalence of fever and malaria infection. To allow direct comparison of results from this study from 2010 to 2012, with the previous study from 2004 to 2008 [15, 40], only data from the original 15 city centre wards common to both studies (Fig. 1) were included and summarized by survey round. **a** Schematic summary of specific intervention introductions; **b** Stayed in a ward with larvicide application (LA) last night; **c** Stayed in a house with mosquito-proofed windows, ceilings or eaves; **d** Used a bed net or long-lasting insecticidal nets (LLIN) the previous night; **e** Treated with an artemisinin-based therapy, including artemisinin-based combination therapy (ACT), or with any other anti-malarial, if had a fever in the previous 2 weeks; **f** Outdoor rates of human exposure to biting malaria vectors; **g** Outdoor rates of human exposure to infectious bites by malaria vectors; **h** Prevalence of reported fever and parasitologically-confirmed malaria infection

**Fig. 3:**
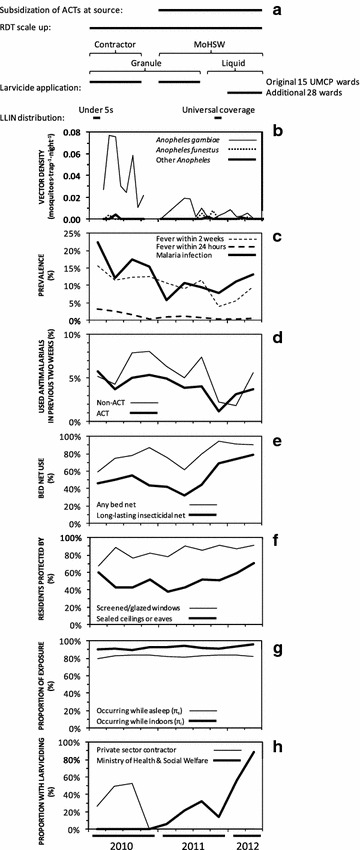
Short-term trends in coverage with malaria control interventions across the city of Dar es Salaam over the course of this study, summarized by quarterly mean. **a** Schematic summary of specific intervention introductions; **b** Vector densities; **c** Prevalence of *Plasmodium falciparum* infection and recollection of fever; **d** Anti-malarial drug use; **e** Bed net use the previous night; **f** Stayed in a mosquito-proofed house; **g** Proportion of potential human exposure to biting vectors expected to occur while asleep or indoors in the absence of protective bed nets or mosquito-proofed housing; **h** Stayed in a ward with larvicide application (LA) last night

The way in which the overall intervention scenario in Dar es Salaam evolved over the course of the study is narrated below, summarized graphically in the context of the long-term intervention history of the city in Fig. 2a, and illustrated in detail in Fig. 3a.

### Drugs

At the outset of the study, subsidies for ACT in Tanzania were restricted to young children and pregnant women, so uptake remained poor across the country generally [39], and in Dar es Salaam specifically [40]. It is therefore unsurprizing that most residents predominantly relied upon private sector health facilities and drug outlets to access anti-malarials [39, 40]. The subsidy of ACT at source, to improve availability through such private sector outlets, was introduced nationally at the start of 2011 and rapidly achieved improved levels of availability and affordability, even in remote rural areas [41–43]. Nevertheless, at least a third of all malaria therapies used in all surveyed rural areas remained non-ACT [41, 43, 44].

### Diagnostics

While microscopic diagnosis of malaria has been available at many health facilities across Dar es Salaam for decades, routine standards of practice were very poor, with gross levels of overdiagnosis consistently observed across all levels of facilities [45, 46]. Country-wide scale-up of rapid diagnostic tests (RDT) for malaria began in 2010, following a successful pilot in Dar es Salaam [46–48], but has so far only been evaluated in rural [49] or semi-urban areas [50].

### Larval source management

Impressive historical successes with larval source management programmes, both before and after independence [27–36], were recently succeeded by an integrated operational research programme, which demonstrated the effectiveness and affordability of both environmental management [51, 52] and routine LA [15, 40, 53], as implemented, monitored and evaluated through contemporary, community-based strategies [12, 13, 23, 24, 54–57]. In addition to pilot-scale evaluations of environmental management spanning 2006 and 2007 [51, 52], new systems for implementing, monitoring and managing LA using microbial insecticides were developed and established in three urban wards by May 2006, and then steadily scaled up to encompass 15 urban wards, 55 km^2^ and 610,000 residents by early 2008 [12, 13, 15, 16, 23, 24, 54, 55].

Following completion of this operational research phase of the Dar es Salaam Urban Malaria Control Programme, the MoHSW of mainland Tanzania decided in 2009 to absorb LA activities into its National Malaria Control Programme (NMCP), but this institutionalization process was not completed until the end of 2010 [13]. In the interim, between January 2009 and October 2010, these LA activities were implemented in the same 15 wards covered in the original operational research phase of the programme (Fig. 1), temporarily under the direct management of a private sector contractor (Research Triangle International) during its last year of external funding support [58]. Once institutionalization of the programme management into the MoHSW had been completed at the end of 2010, LA was re-initiated in these same 15 wards in January 2011 and sustained until July of that year when stocks of *Bti* were exhausted. Up to this point, the larvicide product used was a WHOPES-recommended *Bacillus thuringiensis* var. *israelensis* (*Bti*) product (VectoBac^®^, Valent BioSciences Corporation), predominantly in the form of a coated corn cob granule formulation, but also occasionally water dispersible granules. From August 2011 onwards, aqueous suspension formulations of an alternative, non-WHOPES-recommended *Bti* product (Bactivec^®^, LABIOFAM^®^) was used. LA with this products was then scaled up to 28 additional neighbouring wards and sustained into 2015.

### Mosquito-proofing houses

As improved, more appropriate and affordable construction materials became available on the open market in Dar es Salaam, protection of houses against mosquito entry with window screening, and with closed ceilings or eaves, steadily increased between 2004 and 2008, despite the absence of any programme to subsidize or promote these measures [14, 40]. Residents cited protection against mosquitoes as their primary motivation for investing in these housing improvements [14] and spent more time indoors in the evenings if both measures were in place [9]. Increasing uptake of these measures was associated with a steady decline in population-wide malaria prevalence and interacted synergistically with roll out of LA [15].

### Long-lasting insecticidal nets

Insecticide-treated nets (ITNs) and then LLINs were the standard first-priority vector control measure in Tanzania throughout this study. The Tanzanian National Voucher Scheme was then initiated in 2004, to subsidize purchase of ITNs through a discount voucher provided to pregnant women seeking ante-natal care and mothers of young children when they are vaccinated [59]. However, progress with scale-up of LLINs to full universal coverage targets lagged behind that for LSM and house screening in Dar es Salaam. The city has been consistently classified as having relatively low levels of transmission and, therefore, correspondingly given the lowest priority in national “catch up” programmes for free net distribution. Dar es Salaam was the last region of Tanzania to be reached by the mass distribution to children under the age of 5 in early 2010 [60], and by the subsequent campaign to achieve universal coverage of remaining sleeping spaces in late 2011 [61].

### Data collection procedures

#### Cross-sectional household surveys of malaria infection prevalence and sero-prevalence

The total number of households in each TCU within the mapped study area [24] was enumerated by census between January 2008 and May 2010. While awaiting completion of this enumeration of 543209 households in the city, a first phase of purposively-sampled household surveys were conducted. This first survey phase consisted of a single survey round of 264 housing compounds, 156 of which were also subjected to longitudinal, community-based surveys of vector density on a monthly basis between March and September 2010, so that this novel system for monitoring vector densities at high levels of spatial resolution could be evaluated in terms of its epidemiological predictive power [56]. Following completion of the household listing, a second phase of household surveys were conducted, beginning with a second survey round between October 2010 and September 2011 that sampled much smaller numbers of larger population clusters, selected randomly as index TCUs in proportion to their estimated population size. A total of 68 index TCUs were randomly selected with probabilities weighted according to the number of household recorded for each by the household enumeration surveys. In each index TCU, households were listed afresh and 20 were randomly selected for cross-sectional surveys. Where the TCU had less than 20 consenting household heads, a neighbouring TCU was chosen at random, from where the remaining number of households required were selected and recruited in the same way. The second phase was then extended with a third round of cross-sectional surveys, carried out from October 2011 to May 2012, which repeated the second round of surveys in 28 of the originally sampled clusters and 17 new ones in cases where the TCU leaders of the clusters sampled in round 2 indicated unwillingness to participate in the follow up surveys.

All consenting and assenting household members of all ages, except for children of 3 months or less, were surveyed with a standardized questionnaire and RDT for *P. falciparum* Histidine Rich Protein 2 (HRP-2) antigenaemia in peripheral finger-prick blood samples, as previously described in detail [56, 62]. Individuals found to be infected were treated with artemether–lumefantine according to national guidelines of the MoHSW. Those with a negative RDT result who exhibited symptoms of illness were referred to the nearest public-sector health facility for examination, diagnosis and, where appropriate, treatment of the most likely cause(s) of disease. RDT cassettes were then stored at −20°C and subsamples of 1134 and 345 of the strips within them were eluted and tested for sero-reactivity to *P.falciparum* apical membrane antigen (AMA) [63] and the presence of parasite DNA by polymerase chain reaction (PCR) [64], respectively, as previously described.

#### Entomological surveys of mosquito densities and behaviour

The community-based system for using Ifakara tent traps (ITTs) [65] to continuously monitor vector densities that was initially developed and sustained until October 2010, when funding ran out, has been described in detail [56]. This system was reinitiated, inclusive of quality assurance surveys with both HLC and ITT by a carefully supervised team of specialist technicians, in May 2011 when additional funding was secured. This surveillance platform was then steadily scaled up by the end of 2011, to encompass monthly vector density measurements at 1062 locations across the entire study area, including 79 of the 85 population clusters for the second and third rounds of cross-sectional household surveys. These scaled-up mosquito surveys, spanning the entire city, were sustained until January 2013. In addition to these longitudinal surveys of vector population dynamics, a sequence of cross-sectional human landing catch surveys were conducted as components of a variety of studies of various mosquito traps and malaria prevention measures [9, 10, 66–69], so that the feeding behaviours of local vector populations were regularly characterized throughout the study period. All *Anopheles* captured were tested for the presence of *P.falciparum* circumsporozoite protein by enzyme-linked immunosorbent assay [70], including confirmation by re-assay after heating homogenates of apparently positive samples [71]. All specimens from the *An. gambiae* complex were also tested for species identity by PCR [72].

### Statistical analysis

All statistical analyses were conducted with IBM^®^ SPSS^®^ and Microsoft^®^ Excel^®^ to prepare and conduct descriptive analysis of the data, as well as R software for fitting generalized linear mixed models (GLMMs).

Apart from descriptive summaries with tables and frequency distributions, all analyses of *P. falciparum* malaria infection prevalence were accomplished by fitting generalized GLMMs with a logit link function and binomial distribution for this binary dependent outcome, and including date and household nested within sample cluster as independent variables with random effects, using the *lme4* package. For all independent variables collected as continuous numbers and categorical variables with more than two possible values, initial exploratory analysis of their effect on malaria prevalence were conducted to establish how best to stratify or combine values for inclusion in more complex models with multiple variables. This same exploration and simplification approach was also applied to complex interactions between two or more variables, such as window screens, eaves, ceilings and LLINs. Surveyed human subjects were only considered to have possibly experienced potential benefits of LA if this intervention had been implemented in that ward for at least a month before the individual was interviewed and tested.

Given the clear evidence of outdoor transmission as a potential risk factor for malaria infection in Dar es Salaam [9, 10], the role of each surveyed individual’s reported behaviours was evaluated by calculating and stratifying individual estimates [73] for the proportion of exposure to bites by malaria vectors that would occur indoors (*π*_*i*_) or during sleeping hours (*π*_*s*_) in the absence of any personal protection interventions like bed nets or mosquito-proofed housing [74–76]. Estimates for these two indicators of potential for exposure while outdoors (*π*_*i*_) and awake (*π*_*s*_), were calculated for each individual based on their specific responses to questions about their behaviour in that particular survey [73]. This was accomplished with the simple binary approach to classifying each individual as being indoors or asleep, which was previously described for entire populations based on their median responses [74–76], using a customized Excel^®^ spreadsheet (Additional file 1). The estimates used for the analyses presented here were made based on mean observed indoor and outdoor biting densities of *An. gambiae s.l.* for each hour of the night during intensive HLC surveys in 2006 [9, 10], before these cross-sectional household surveys commenced in 2010. However, all these analyses were also attempted with species-specific measurements based on PCR-identified *An. gambiae* sensu stricto or *An. arabiensis* alone captured in the same 2006 experiments [9, 10], or with equivalent HLC data from similarly intensive mosquito trap evaluations conducted in 2010, but none of these improved the goodness-of-fit statistic for relevant analytical models and had less precise estimates because of lower numbers of mosquitoes caught after vector populations had been suppressed by programmatic roll out of LA.

The impacts of LA upon densities of common mosquito taxa in the city were estimated using the *lme4* package by fitting GLMMs with negative binomial distributions to the counts of mosquitoes caught by each catcher on each night at a given location with an ITT as the dependent variable. LA for at least 2 weeks before the night of survey was included as a fixed effect, while TCU nested within ward was treated as random effects.

Longitudinal surveys of vector density were not active throughout all rounds of the cross-sectional household surveys of parasites amongst humans between 2010 and 2012: A break in funding support for this platform occurred between November 2010 and April 2011. Results from the first set of vector surveys that exactly matched to the locations and duration of the first round of cross-sectional parasite surveys were aggregated to generate mean trap catch estimates for each of the 156 co-surveyed TCUs to which these values were linked. Similarly, the subsequent entomological surveys that straddled the second and third round of cross sectional surveys, which used a different sampling frame, were aggregated to generate mean trap catches that were linked to the epidemiological data from the same 79 TCUs in one or both rounds of parasite surveys.

### Ethics, consent and permissions

The study received ethical clearance from the Medical Research Coordination Committee of the Tanzanian National Institute of Medical Research (Reference numbers NIMR/HQ/R.8a/Vol.IX/279 and 324). Informed consent was obtained from all the participants, including the mosquito catchers and the house owners where the sampling took place, as well as the participants in the household surveys. All the volunteers recruited for conducting HLC were provided with prophylactic treatment with atovaquone–proguanil (Malarone^®^) free-of-charge, which they were obliged to take once a day to prevent malaria infection. In order to deal with the possibility of poor compliance or drug failure, participants in mosquitoes-trapping surveys who developed any symptoms such as fever, chills, headache or nausea, were tested for malaria parasites. They would have been offered free treatment if found to be infected, but this eventuality never occurred during the study. All participants in the household surveys who were found to be infected with malaria were offered supervised treatment with artemether–lumefantrine (Coartem^®^; Novartis Pharma AG, Basel, Switzerland), prescribed by a clinical officer and provided by the community health nurse, following national treatment policies and guidelines, as soon as the RDT test was complete. However, if the participant refused this offer of treatment, they were referred to a nearby health facility and given all required transport and other logistical assistance to attend. Women of child-bearing age who were found to be infected with malaria were offered treatment with artemether–lumefantrine unless they were known or suspected to be pregnant and in their first trimester, in which case they were instead treated with oral quinine as per national guidelines.

## Results

### Area-wide trends in malaria transmission and human infection prevalence

During the first phase of cross-sectional household surveys, at the outset of this study in 2010, mean malaria prevalence across the city appeared far higher (13.8 %) than in 2008 (1.7 %), at the end of an operational research programme during which LA was comprehensively scaled up to 15 wards in the city centre [12, 15, 40]. Restricting analysis, to the 15 central wards which were common to both studies, confirmed this return to high levels of malaria transmission hazard (Fig. 2f, g) and infection prevalence (Fig. 2h). This rise in malaria prevalence occurred despite continued financial support for LA across all 15 of these wards (Fig. 2b), sustained high coverage of mosquito-proofed housing (Fig. 2c), and LLIN usage almost doubling following free mass distribution to under five children [60] in early 2010 (Fig. 2d), and slightly increased proportions of fevers being treated with ACT (Fig. 2e).

This resurgence of malaria prevalence, following transfer of LA management to a private contractor in 2009 and 2010, is consistent with an observed rebound in *An. gambiae* densities (Fig. 2f, g), which prevalence trends generally tracked reasonably consistently over the long term (Fig. 2h). It is also consistent with the lack of any obvious association between LA and either vector density (Table 1) or infection prevalence (Table 2) in 2010. While the estimate of 3.7 infectious bites·person^−1^ year^−1^ for the EIR mediated by *An. gambiae* in 2010 is undoubtedly biased upwards, because these surveys were restricted to a few months of the year with peak vector densities, this is nevertheless much higher than estimates for 2008 after LA had been scaled up (0.8 infectious bites·person^−1^ year^−1^), and even than before LA was introduced in 2005 (2.1 infectious bites·person^−1^ year^−1^) (Chaki et al., Unpublished).

**Table 1:** Factors affecting numbers of *Anopheles gambiae* malaria vectors caught in Ifakara tent traps over 12,170 trap nights of capture in 1562 locations distributed across Dar es Salaam, Tanzania

Variable	Proportion (n)	Odds ratio [95 % CI]	P
*Live in one of the original 15 Urban Malaria Control Programme (UMCP) study wards*			
New study ward	51.0 % (6211)	1.00 [NA]	NA
Old UMCP study ward	49.0 % (5959)	3.36 [2.14, 5.29]	0.033
*Living in a ward with or without active Bti larvicide application*			
No larviciding	38.3 % (4664)	1.00 [NA]	NA
Granule application managed by contractor^a^	19.9 % (2416)	0.83 [0.16, 4.2]	0.820
Granule application managed by Ministry of Health & Social Welfare (MoHSW)	5.9 % (717)	0.31 [0.14, 0.71]	0.0053
Pre-diluted liquid application managed by MoHSW	35.8 % (4373)	0.15 [0.07, 0.30]	0.000000079
*Population*-*wide LLIN use* ^b^			
Increase in coverage by final round	From 51.0 to 71.2 %	0.72 [0.51,1.01]	0.057

*NA* not applicable

^a^Excluded from the final model by merging with reference group because non-significant, but presented here for illustrative purposes

^b^Association with community-level mean LLIN scale-up, captured by fitting city-wide mean reported LLIN use the previous night as a continuous covariate, so the relative rate presented is that estimated based on community-wide usage in the last round of surveys (71.2 %) versus the first (51.0 %)

**Table 2:** Minimal logistic generalized linear mixed model describing risk factors for malaria among 9172 RDT-tested occupants of 2822 households in Dar es Salaam, Tanzania, surveyed between March 2010 and May 2012, for whom valid values of all significant variables were recorded

Variable	Proportion (n)	Odds ratio [95 % CI]	P
*Live in one of the original 15 Urban Malaria Control Programme (UMCP) study wards*			
New study ward	68.8 (7693)	1.00 [NA]	NA
Old UMCP study ward	31.2 (3494)	1.30 [1.02, 1.65]	0.033
*Education of head of household*			
Any	96.8 % (8882)	1.00 [NA]	NA
None	3.2 % (290)	1.90 [1.34, 2.71]	0.00035
*Sex*			
Female	63.9 % (5864)	1.00 [NA]	NA
Male	36.1 % (3308)	1.16 [1.03, 1.33]	0.020
*Window screening* ^a^ *and the proportion of potential vector biting exposure occurring indoors (π*_*i*_ *)* ^b^			
Unscreened or screened but lowest *π*_*i*_ tercile	34.1 % (3132)	1 [NA]	NA
Screened and middle or highest *π*_*i*_ tercile	65.9 % (6040)	0.71 [0.62, 0.82]	0.0000036
*Living in a ward with or without active larvicide application*			
No larviciding	69.2 % (7744)	1.00 [NA]	NA
Granule application managed by contractor^c^	11.9 % (1329)	1.29 [0.78, 2.13]	0.325
Granule application managed by Ministry of Health & Social Welfare (MoHSW)	4.9 % (551)	0.26 [0.12, 0.56]	0.00040
Pre-diluted liquid application managed by MoHSW^c^	14.0 % (1563)	0.96 [0.67,1.37]	0.836
*Individual use of a bed net the previous night*			
Didn’t use any bed net	19.4 % (1773)	[NA]	NA
Used an untreated bed net	29.8 % (2736)	1.29 [1.03, 1.60]	0.023
Used a long-lasting insecticidal net (LLIN)	50.8 % (4663)	1.42 [1.16, 1.74]	0.00063
*Population*-*wide LLIN use* ^d^			
Increase in coverage by final round	From 51.0 to 71.2 %	0.80 [0.69, 0.91]	0.0013
*Recent travel history over last month*			
Had not slept away from home	79.8 % (7322)	1.00 [NA]	NA
Slept away from home at least once	20.2 % (1850)	0.69 [0.54, 0.88]	0.0024
*Anopheles gambiae density* ^e^ *among households lacking window screening* ^a^ *only*			
One mosquito caught per trap per night	Continuum	6.99 [1.12, 43.7]	0.037
*Age group* ^c^			
Under 5 years	16.7 (1873)	1.00 [NA]	NA
5–14 years	18.4 (2062)	1.06 [0.86, 1.32]	0.542
15–24 years	22.6 (2526)	1.16 [0.95, 1.44]	0.143
25–34 years	18.2 (2040)	0.97 [0.77, 1.21]	0.776
35 years and above	24.0 (2686)	1.01 [0.82, 1.24]	0.947

*NA* Not applicable

^a^Based on exploratory analysis as described in the main text, with glass, completely screened with no holes and completely screened with holes classified as adequately-screened and protective, whereas unscreened, torn or only partially screened were all classified as inadequately screened

^b^See Fig. 8 for a description of the behavioural characteristics of the three terciles of this index of the proportion of human exposure to mosquitoes which occurs indoors and can be prevented with indoor vector control measures [74, 75, 106–109]

^c^Excluded from the final model because non-significant, but presented here for illustrative purposes

^d^Association with community-level mean LLIN scale-up, captured by fitting city-wide mean reported LLIN use the previous night as a continuous covariate, so the odds ratio presented is that estimated based on community-wide usage in the last round of1 surveys (71.2 %) versus the first (51.0 %)

^e^Presented here for illustrative purposes but excluded from the final mode, because reducing vector density is an intermediate outcome of both bed net use and larvicide application, so inclusion confounds evaluation of these vector control measures. Incorporated into the model as a square root-transformed continuous variable so the odds ratio presented represents that estimated for any area with an *Anopheles gambiae* density of one mosquito per tent trap per night compared with a location where none were detected by tent trapping. To get this threshold in context, the highest density of *An. gambiae* recorded was only slightly lower than this (Fig. 6)

**Fig. 4:**
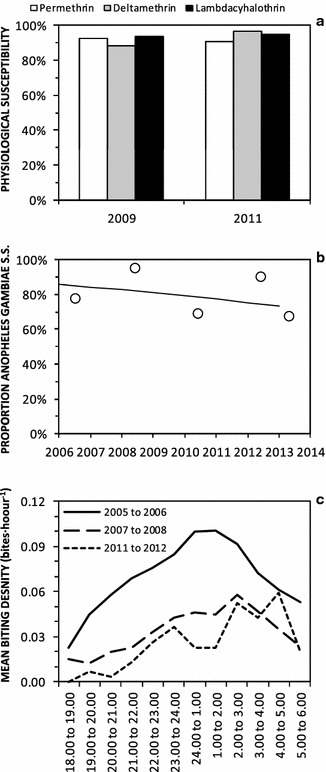
Time trends in physiological susceptibility to pyrethroids (**a**), sibling species composition (**b**), and biting activity distribution (**c**) of *Anopheles gambiae* sensu lato in Dar es Salaam. Physiological susceptibility estimates (**a**) were obtained from published surveys [110]. Sibling species composition data (**b**) were obtained from PCR analysis of mosquitoes caught through both the routine surveillance collections described here and a range of published [9, 10, 66, 68] and unpublished experimental studies of trapping methods conducted at intense sampling in foci of high vector density. Biting activity distribution data (**c**) were obtained from outdoor HLC data obtained through either routine surveillance from 2005 to 2008, or through quality assurance surveys of routine CB mosquito trapping with Ifakara tent traps between 2011 and 2012

Perhaps even more surprising is the observation that infection prevalence of approximately 10 % persisted throughout the study (Fig. 3c), despite sustained coverage of LA in 2010 and 2011, followed by scale up in 2012 (Fig. 3h), rapid upscale of LLINs from the end of 2011 onwards (Fig. 3e), and continuing progress with uptake of window screening (Fig. 3f), resulting in a dramatic crash in vector population density (Fig. 3b). By the third and final round of surveys, 93 % of participants slept under a net of some kind and 72 % under an LLIN specifically, 80 % slept in houses with mosquito-proofed windows and 57 % in houses with sealed ceilings or eaves, while 41 % lived in wards with ongoing LA (Additional file 2; Fig. 3e, f, h respectively). Despite all this additional intervention pressure upon host seeking adults by mosquito proofed houses and bed nets, the distribution of biting activity of *An. gambiae s.l.* across different times of the night remained approximately consistent with those observed previously, except perhaps for a slight shift to later hours of the night, between midnight and dawn (Fig. 4c). Human behaviour also remained stable throughout the study, with consistently high means for individual-level estimates for the proportions of potential exposure which would otherwise occur indoors (*π*_*i*_) or while asleep (*π*_*s*_) in the absence of any of these interventions (Fig. 3g).

Throughout the study period, only modest levels of physiological resistance to pyrethroids (Fig. 4a), and behavioural resistance or resilience [77] to indoor vector control measures by biting at dawn or dusk (Fig. 4c), were observed for the *An. gambiae s.l.* complex. Over the course of this study, composition of the *An. gambiae* complex was consistently predominated by the nominate sibling species *An. gambiae s.s.*, with the small remainder being *An. arabiensis* (Fig. 4b)*. Anopheles gambiae s.s.* is the most anthropophagic and efficient sibling species from this complex [78], and very few *An. funestus* or other *Anopheles* were caught (Fig. 3b). Vector densities were therefore expressed in terms of mean numbers of *An. gambiae* complex specimens caught per trap per night for subsequent analyses, and considered representative of this dominant, nominate sibling species. While the mean sporozoite prevalence among *An. gambiae* specimens captured over this period (1.3 %, 2/150) was approximately comparable with those observed between 2004 and 2008, these data are far too sparse to determine whether reduced infection rates were also achieved by LLIN scale-up. Approximate estimates of entomologic inoculation rate (EIR) were, therefore, based upon the parsimonious assumption that this sporozoite prevalence estimate was constant throughout the study.

*Anopheles gambiae* densities had declined considerably by 2011, after the MoHSW took over management of LA, using the same granular *Bti* product as the private sector contractor responsible in 2010, resulting in an estimated EIR of 0.5 infectious bites∙person^−1^ year^−1^ in the 15 previously-surveyed wards with which historical comparisons may be made (Fig. 2g). *Anopheles gambiae* densities continued to decline, after transition to use of the liquid product from August 2011 onwards was then followed by scale-up of LA from 15 to 43 wards in January 2012, as well as mass distribution and correspondingly increased use of LLINs at the end of 2011 (Fig. 3d, e). This combination of interventions resulted in an estimated EIR of only 0.2 infectious bites∙person^−1^ year^−1^ in 2012, in the 15 previously-surveyed wards with which historical comparisons may be made (Fig. 2g).

Increasing usage of LLINs (Fig. 3e), following a mass distribution campaign to “catch up” on universal coverage targets in late 2011, was associated with modestly reduced vector density (Table 1). Analysis of the cross sectional survey data of infection prevalence amongst humans confirms that, while no personal protection could be demonstrated for use of either untreated or treated nets (indeed both were counter-intuitively associated with increased malaria risk; Table 2), increasing city-wide LLIN use (Fig. 3e) was also associated with correspondingly modest community-level reductions of malaria prevalence (Table 2).

**Fig. 5:**
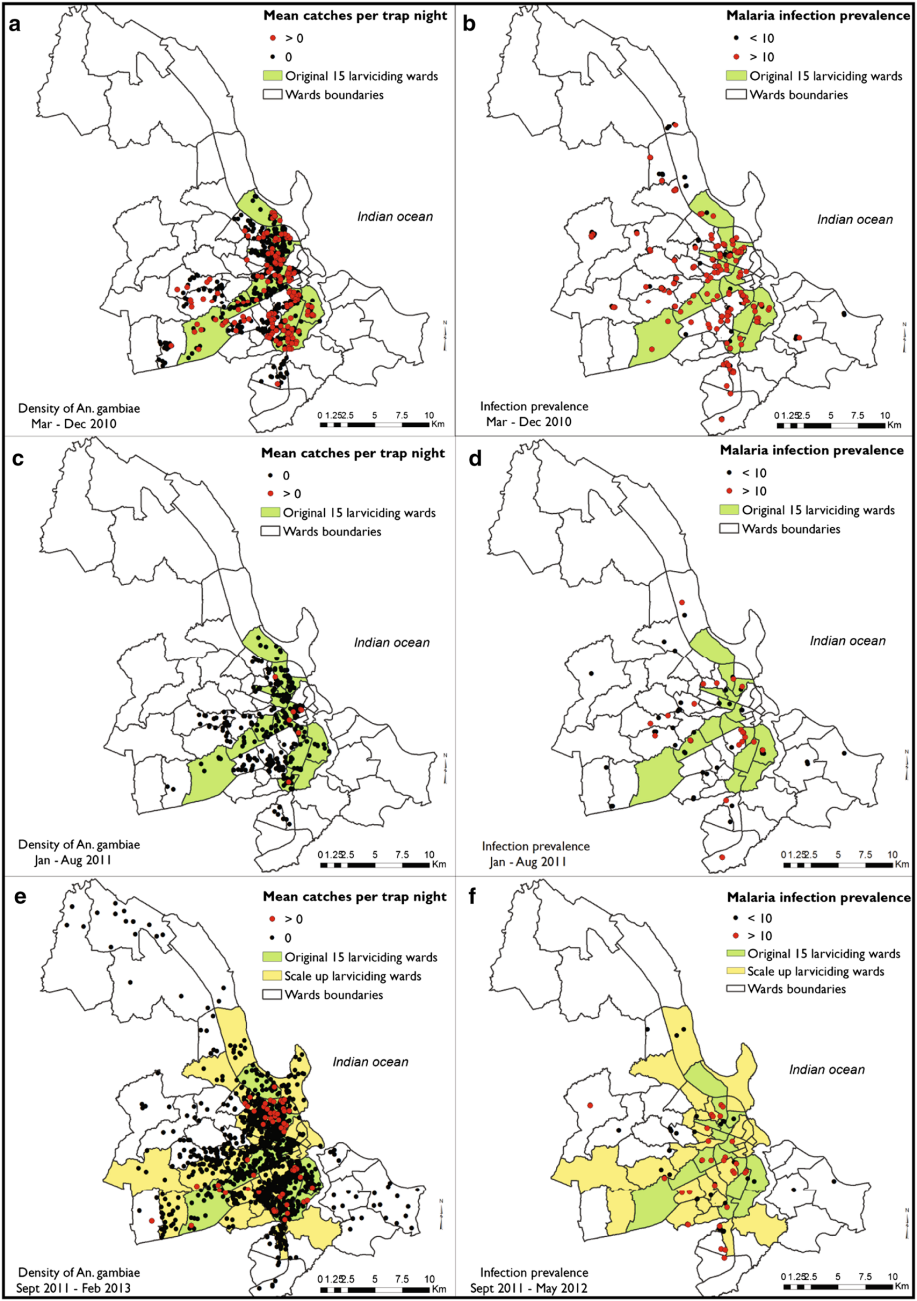
The geographic distribution of surveyed locations with detectable populations of *Anopheles gambiae* vectors (**a**, **c**, **e**) and > 10 % *Plasmodium falciparum* infection prevalence (**b**, **d**, **f**) over the periods from March to December 2010 when application of a granular formulation of *Bacillus thuriniensis *var. *israelensis* (*Bti*) in the wards highlighted in *green* was managed by a private sector contractor (**a**, **b**), from January to August 2011 when the same granular formulation was applied under management of the Ministry of Health and Social Welfare (MoHSW) in the same wards highlighted in *green * (**c**, **d**), and from September 2011 onwards when a pre-diluted liquid formulation of *Bti* was applied under MoHSW management in the wards highlighted in *green* and *yellow* (**e**, **f**)

LA had no apparent impact upon vector density (Table 1; Fig. 5a) or human infection prevalence (Table 2; Fig. 5b) while directly managed by a private-sector contractor using the granular *Bti* formulation. However, following transition to MoHSW management at the start of 2011, it was consistently associated with dramatically reduced local vector densities, regardless of whether the granule or liquid product was used (Table 1), with no difference in apparent effect between the two formulations (P = 0.208). The obvious effects of MoHSW-managed application of the granular *Bti* product upon vector density in the first half of 2011 (Table 1; Figs. 3b, 5c) were matched by corresponding reductions of human infection prevalence (Table 2; Figs. 3c, 5d). However, after MoHSW switched to the liquid product in the second half of 2011, similarly impressive reductions of vector density (Table 1; Figs. 3b, 5e), were no longer associated with corresponding epidemiological impact upon infection prevalence, which remained static into 2012 (Table 2; Figs. 3c, 5f).

**Fig. 6:**
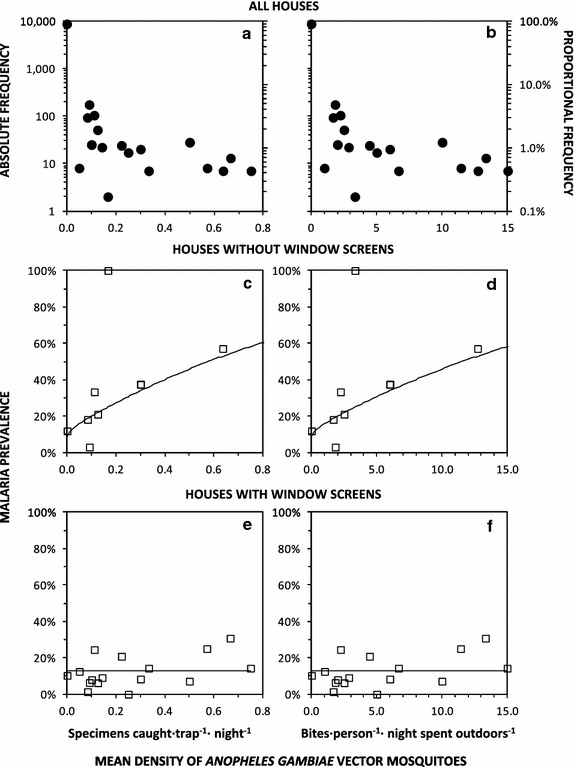
The frequency distribution and dependence of *Plasmodium falciparum* malaria prevalence upon densities of *Anopheles gambiae* sensu lato in Dar es Salaam. The number and proportion (**a**, **b**) of RDT-tested human subjects, as well as the proportion of those which were diagnosed as infected with malaria in houses with (**e**, **f**) and without (**c**, **d**) window screening, are plotted against vector density, as measured by community based surveillance with Ifakara tent traps (**a**, **c**, **e**) and converted into the estimated equivalent outdoor human landing catch (**b**, **d**, **f**). Continuous lines represent the best fit of models relating malaria infection prevalence to vector density in houses with (**c**, **d**) and without (**e**, **f**) window screens

While the methods used to measure both human infection prevalence and vector density differed from those used by the preceding studies [15, 16], with which they are compared in Fig. 2f, g and h, careful examination of quality-assurance indicators for these new survey tools do not suggest any reason to doubt the authenticity of the apparent resurgence of malaria in 2010 and sustained high prevalence in 2011 and 2012. Out of the small subsample of 345 participants whose RDT test strip was also tested for the presence of *P.* *falciparum* DNA by PCR, infection was confirmed in more than two-thirds (68.5 %; 37/54) of those found to be antigen-positive by RDT. Similarly, entomological surveys through community-based application of tent traps correlated well with the quality-assurance HLC surveys, were at least as sensitive in terms of absolute numbers of mosquitoes caught, and are correlated with local malaria infection prevalence [56], albeit only in fully-exposed houses lacking protective window screens (Fig. 6c, d; Table 2).

### Household and individual risk factors for malaria infection

**Fig. 7:**
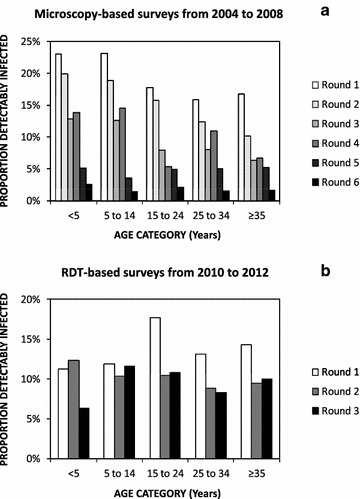
Age-prevalence profiles for *Plasmodium falciparum* malaria infection observed by microscopy in previous cross-sectional surveys from 2004 to 2008 [15] (**a**) and by rapid diagnostic test in surveys during these subsequent surveys between 2010 and 2012 (**b**)

Across all three survey rounds from 2010 to 2012, malaria risk was higher among males and individuals whose household head lacked any education (Table 2). In stark contrast to the previous studies, before most of these interventions had been fully scaled up, no strong variation in RDT-detected prevalence across age groups was obvious (Fig. 7; Table 2).

Despite this apparent loss of immunity (Fig. 7; Table 2), substantive malaria prevalence persisted, simply because the vast majority of infected participants (88 % (1090/1237) had not experienced fever recently, and only 0.37 % (4/1090) of these chronic carriers had been treated for malaria. By the end of the study (Fig. 3c; Additional file 2), the final round of surveys reveals improved rates of treatment of fevers (144 cases) with anti-malarial drugs (91 %; 131 cases), but that use of ACT specifically remained mediocre (42 %; 62 cases). Recent, routine treatment, with neither an ACT (P = 0.984) nor any other anti-malarial drug (P = 0.862), had any detectable effect upon risk of RDT-detected malaria antigenaemia, presumably because drug treatment is an outcome of malaria infection, rather than vice versa, and the antigenaemia which these RDTs detect can last 2 weeks after termination of infection [79].

As observed previously in this setting [15], having stayed elsewhere over the previous month was associated with reduced malaria infection probability (Table 2). Re-analysis of the legacy data from previous studies [15] not only confirmed this observation, but also allowed disaggregation of those who slept elsewhere in Dar es Salaam from those who had slept outside of the city. No difference in risk between travel within and outside the city was apparent (P = 0.331), with a similar trend towards reduced risk in the former [OR (CI) = 0.88 (0.77, 1.02), P = 0.082] and the latter [OR (CI) = 0.77 (0.58, 1.02), P = 0.071], relative to those who had not slept elsewhere. Travel history, therefore, seems unlikely to be a major contributor to ongoing exposure and persistent high prevalence at the end of the study, so most malaria cases were probably locally acquired within the city.

Even more surprisingly, using a bed net was counter-intuitively associated with increased, rather than decreased, malaria risk (Table 2). Of course an equally valid question is whether bed net use behaviour is stimulated by experience of malaria infection, rather than vice versa. Fortunately, RDT eluates from a subsample of 1134 subjects were tested serologically for evidence of a history of previous exposure to malaria AMA antigen. In this subset of subjects, use of a bed net the previous night was positively associated with AMA-reactive serum [OR (95 % CI) = 1.67 (1.04, 2.69), P = 0.034] but not with infection at the time of the survey (P = 0.681), indicating that past experience of malaria infection probably motivates bed net use, rather than immediately active but presumably chronic infection at the time of the survey. Reassuringly, however, increased community-wide LLIN use was associated with substantial reductions of malaria vector density (Table 1) and infection prevalence amongst residents (Table 2).

In contrast to previous studies [15], window screening that was complete but had holes appeared to provide similar levels of protection to complete screening without holes [OR (95 % CI) = 0.74 (0.57, 0.97), P = 0.028 versus OR = 0.89 (0.69, 1.13), P = 0.339, respectively, and P = 0.096 when compared with each other], so these were combined into a single category in the final fitted model (Table 2). Stratified analysis revealed that local vector density was only predictive of increased malaria risk [OR 95 % CI = 22.5 (2.7, 186.2), P = 0.0039] among the minority of participants (14 %; 1308 RDT-tested occupants from 347 households) staying in houses lacking window screening (Fig. 6c, d). No effect of local vector density was observed (P = 0.428) among the majority of participants (86 %; 8036 RDT-tested occupants of 2492 households) staying in houses with window screening (Fig. 6e, f). The final model using data from all households therefore only considers vector density conditional upon lack of window screens (Table 2).

The estimated proportion of exposure to *An. gambiae* bites that would occur while asleep in the absence of a bed net or window screening (*π*_*s*_) had no apparent influence upon infection probability among all residents (P = 0.456), and among those living in houses with (P = 0.468) or without (P = 0.576) window screening. However, high values for the estimated proportion of exposure to *An. gambiae s.l.* bites that would occur indoors in the absence of a bed net or window screening (*π*_*i*_) was protective against malaria risk, but only for the majority of participants who lived in well-screened houses (P = 0.00832), and not those living in unscreened houses (P = 0.792). More careful examination revealed no apparent difference in malaria risk between the upper and middle terciles of this behavioural metric of maximum potential for indoor protection among participants with window screening [OR (95 % CI) = 0.71 (0.59, 0.86), P = 0.000304 and 0.72 (0.57, 0.89), P = 0.00327] for highest and middle terciles versus the lowest, respectively, and P = 0.970 for the contrast between the middle and high strata), or between the lowest tercile living well-screened houses versus those living in unscreened houses (P = 0.611). The final model using data from all households therefore captures the interaction between window screens and human behaviour in terms of one high risk category and one low risk category: Lack of complete window screening or well-screened windows but lowest *π*_*i*_ tercile versus well-screened windows and middle or upper *π*_*i*_ tercile (Table 2).

**Fig. 8:**
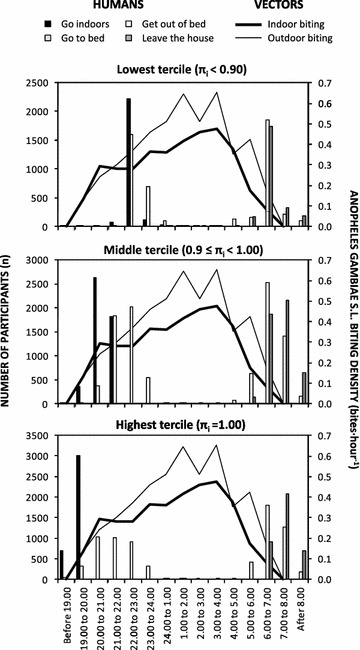
Times at which individuals interviewed during cross-sectional household surveys in Dar es Salaam reported having gone indoors for the evening, gone to bed for the evening, gotten out of bed in the morning and left the house in the morning, the previous night, stratified by derived individual estimates for the proportion of exposure to *An. gambiae* bites that would occur indoors in the absence of a bed net or window screening (*π*_*i*_). For comparison with the biting activity profile of the most important malaria vector in the city, these frequencies of human behaviours are plotted alongside the human biting rates measured by human landing catch (HLC) in selected areas of relatively high vector density in 2006 [9, 10] that were used to calculate these individual estimates for *π*_*i*_

Of all the contributing human behaviour variables used to estimate the proportion of exposure to *An. gambiae s.l.* bites that would occur indoors in the absence of a bed net or window screening (*π*_*i*_), none were predictive of malaria risk in their own right. Neither the time at which residents went indoors (P = 0.413) and then went to bed in the evening (P = 0.583), or got out of bed (P = 0.198) and then left the house in the morning (P = 0.121) had any obvious influence upon malaria infection risk. However, examining the distribution of values reported for all these variables reveals remarkably subtle differences between these epidemiologically-relevant behavioural strata: Few participants stayed outdoors beyond 23.00 h or left the house before 05.00 h (Fig. 8), so it seems that even a few extra hours of outdoor exposure in the evenings or mornings result in comparable malaria risk to spending all night in a house with no window screens.

## Discussion

The greatest strength and weakness of this study is its purely observational nature, because no effort was made to experimentally control routine, programmatic delivery of any malaria control measure. Observational studies are inevitably prone to biases [80], so this study only provides evidence of *plausibility* rather than *probability* [81]. However, observational studies also have the advantage of being highly relevant to policy and practice if they are conducted by an independent evaluation team and the interventions themselves are delivered under representative conditions of routine programmatic practice [81]. All these surveys of malaria prevalence and mosquito densities were conducted by an independently-funded research team at the Ifakara Health Institute, completely independently of the UMCP staff based at either the municipal councils of Dar es Salaam or at the NMCP of the MoHSW [13]. So, unlike the impact evaluations conducted in the operational research phase of the UMCP between 2004 and 2008 [12, 15, 40], during which the research team was actively involved in planning, delivering and monitoring the LA intervention, this study can be described as a fully representative assessment of programmatic effectiveness [81] of all the interventions implemented in Dar es Salaam over this period.

The most important African malaria vectors are historically notorious for primarily feeding on humans while they are asleep indoors [75], so outdoor exposure may be of minor epidemiological importance in African settings where predominantly indoor transmission persists [73]. Nevertheless, several recent studies from across the continent with only entomological outcomes have suggested cause for concern [82–84] and this study demonstrates unambiguously that outdoor exposure is not only epidemiologically significant in this typical African city, but also varies according to individual human behaviour patterns (Table 2; Fig. 8).

The observation that even spending one or two extra hours outside of well-screened houses, by going indoors just after 22.00 h and 23.00 h rather than before 22.00 h, or by leaving the house just before 06.00 h rather than afterwards, increases malaria risk to a level equivalent to living in a house without window screening is surprising. It is not obvious why most of the increment risk of malaria infection associated with outdoor exposure appears to occur within such a narrow temporal window, but the brevity and predictability of this exposure window does suggest that time-targeted behavioural and/or protective interventions may be quite feasible. Topical repellents [85], vapour-phase insecticides [69, 86, 87], or insecticidal clothing [88–90] may, therefore, have important applications to prevent residual malaria transmission occurring outside of mosquito-proofed houses and sleeping spaces in Dar es Salaam, and many other settings across the tropics.

The surprising observed positive association of personal net use with malaria risk (Table 2) appears to be at least partially explained by human behavioural responses to experience of malaria episodes (Msellemu et al., Unpublished). Furthermore, the clear reductions of vector density (Table 1) and human infection risk (Table 2) associated with improved community-wide uptake of LLINs in the last year of the study strongly suggests that individual-level protective effects do occur but have probably been masked by this reverse causality.

The lack of any obvious entomological (Table 1; Fig. 5a) or epidemiological impact of LA (Table 2; Fig. 5b) when managed temporarily by a private-sector contractor is striking, particularly when contrasted with the apparent reductions of vector density associated with LA under MoHSW management throughout the remainder of the study (Table 1; Fig. 5c), as well as the reductions of infection prevalence in the first half of 2011 (Table 2; Fig. 5d). It is not clear from our data why implementation by the private-sector contractor was so ineffective, and no records of process or coverage indicators could be obtained to examine why this might have been the case. However, this observational evidence of apparently successful implementation of LA under the management of MoHSW is especially encouraging and timely, now that the Government of Tanzania has made a long-term commitment to scale up LA across all major urban centres of the country. However, given the disappointing results while LA was temporarily managed by a private contractor, and inconsistency of the epidemiological and entomological results after MoHSW switched from the granular product to the liquid product in the second half of 2015 (Tables 1, 2; Fig. 5e, f), it is essential that an equally strategic approach is taken to operational research for developing increasingly effective implementation, monitoring and evaluation systems for routine LA.

Nevertheless, the lack of any obvious epidemiological impact of LA with the liquid formulation, and the persistence of remarkably high levels of human infection prevalence from mid 2011 onwards (Table 2; Fig. 5f), despite very clear impact upon entomological transmission metrics (Table 1; Fig. 5e), merits careful consideration. While it is possible that the apparent entomological impacts arose spuriously from the biases inevitably associated with any observational study [80], this seems unlikely based on the consistency with the results for first half of 2011 with the granule formulation. Given just how sparse the vector population became over this period, how low the EIR estimates became, and the lack of evidence for suppression of infection detection by acquired immunity, a more likely explanation is probably the persistence of chronic infections [91] among residents. Indeed the EIR estimates presented, including that of only 0.2 infectious bites·person^−1^ year^−1^ in 2012, were calculated for someone sleeping outdoors all year round, without adjusting for the fact that most residents slept inside mosquito-proofed houses and/or bed nets (Table 2). The actual mean EIR experienced by the average resident was therefore most probably well below the threshold of 0.1 infectious bites·person^−1^ year^−1^, at which malaria transmission may become unstable and prone to elimination [92–94].

In the absence of longitudinal cohort survey data and associated molecular analyses to identify new, recently acquired infections, it is not possible to conclude definitively that the persisting high levels of parasitaemia observed at the end of the study really are predominantly comprised of chronic infections [95, 96]. Furthermore, such surprisingly high prevalence may also at least partially reflect the fact that once immunity wanes in a population (Table 2; Fig. 7), infection prevalence becomes chaotically dynamic and very difficult to interpret over such short time scales [97]. Nevertheless, the vast majority of infected residents in Dar es Salaam did not report having experienced fever recently, and almost none of these apparently afebrile cases were treated for malaria, even though transmission (Figs. 2, 3) and immunity (Fig. 7) have both clearly waned in recent years. Although further declines in immunity might increase the proportion of malaria infections that cause sufficiently acute clinical symptoms to motivate higher utilization rates of testing and treatment services offered passively at health facilities [98], eliminating human-to-mosquito transmission will probably require more pro-active approaches [91, 99]. Untreated *P. falciparum* infections typically last months or years, during which time they are infectious to mosquitoes [99], so infection prevalence can take several years to respond to changes in transmission intensity [100–104]. Given that such persistent sub-acute infections can also be more accurately described as *chronic* than truly *asymptomatic*, because they cause considerable long-term morbidity and mortality [91], population-wide campaigns to treat chronic, sub-acute infections will probably be required to rapidly and decisively bring an end to local transmission in the city.

## Conclusions

Dar es Salaam is a typical African city in many respects, but is also clearly unusually advanced in terms of delivering integrated malaria control programmatically, using several layers of preventative and therapeutic interventions, to the majority of the population through diverse delivery mechanisms. While this programmatically-sustained integrated malaria control programme has clearly achieved dramatic reductions in malaria burden, the persistence of endemic transmission by remarkably sparse populations of efficient vectors, much of which occurs outdoors [9, 10], is equally notable. While additional measures to protect against outdoor transmission exposure [69, 85–90] are clearly desirable, perhaps the most immediate opportunity for progress towards malaria elimination is further scale-up of LA and mosquito-proofed housing, ceilings and closed eaves in particular. However, accelerated elimination of malaria from this urban setting will also probably require active, population-wide mass screen-and-treat or mass drug administration campaigns to cure chronic human infections [91, 99], which can otherwise persist for many years [100–104].

## Additional files

10.1186/s12936-016-1340-4 Excel^®^ spreadsheet template for calculating individual-level estimates for the proportion of exposure of bites by *Anopheles gambiae* sensu lato that would occur indoors or while asleep in the absence of any protective interventions such as window screens or bed nets. This example is populated with a sample of questionnaire data describing the times residents reported having gone indoors for the evening, gone to sleep for the night, woke up in the morning and left the house in the morning, as well as published patterns of vector biting activity as measured by human landing catch in parts of Dar es Salaam with vector densities that were high enough to measure [9, 10].
10.1186/s12936-016-1340-4 Excel^®^ spreadsheet summaries of sampled households and participants.

## Abbreviations

ACT: artemisinin-based combination therapy
AMA: apical membrane antigen
*Bti*: *Bacillus thuringiensis* var. *Iisraelensis*
CI: confidence interval
DNA: deoxyribonucleic acid
GLMM: generalized linear mixed model
EIR: entomological inoculation rate
HLC: human landing catch
HRP: histidine-rich protein
ITN: insecticide-treated net
ITT: Ifakara tent trap
LA: larvicide application
LLIN: long-lasting insecticidal net
MoHSW: Ministry of Health and Social Welfare
NMCP: National Malaria Control Programme
OR: odds ratio
PCR: polymerase chain reaction
RDT: rapid diagnostic test
RR: relative rate
TCU: ten cell unit
UMCP: Urban Malaria Control Programme

## References

[1] Robert V, MacIntyre K, Keating J, Trape JF, Duchemin JB, Warren M (2003). Malaria transmission in urban sub-Saharan Africa. Am J Trop Med Hyg.

[2] Keiser J, Utzinger J, Castro MC, Smith TA, Tanner M, Singer BH (2004). Urbanization in sub-Saharan Africa and implications for malaria control. Am J Trop Med Hyg.

[3] Hay SI, Guerra CA, Tatem AJ, Atkinson PM, Snow RW (2005). Urbanization, malaria transmission and disease burden in Africa. Nat Rev Microbiol.

[4] Killeen GF, McKenzie FE, Foy BD, Schieffelin C, Billingsley PF, Beier JC (2000). A simplified model for predicting malaria entomologic inoculation rates based on entomologic and parasitologic parameters relevant to control. Am J Trop Med Hyg.

[5] Smith DL, McKenzie FE (2004). Statics and dynamics of malaria infection in *Anopheles* mosquitoes. Malar J..

[6] Keating J, MacIntyre K, Mbogo C, Githeko A, Regens JL, Swalm C (2003). A geographic sampling strategy for studying relationships between human activity and malaria vectors in urban Africa. Am J Trop Med Hyg.

[7] Fillinger U, Lindsay SW (2011). Larval source management for malaria control in Africa: myths and reality. Malar J..

[8] WHO. Larval source management-A supplementary measure for malaria vector control: An Operational Manual. Geneva: World Health Organization; 2013.

[9] Geissbuhler Y, Chaki P, Emidi B, Govella NJ, Shirima R, Mayagaya V (2007). Interdependence of domestic malaria prevention measures and mosquito-human interactions in urban Dar es Salaam, Tanzania. Malar J..

[10] Govella NJ, Okumu FO, Killeen GF (2010). Insecticide-treated nets can reduce malaria transmission by mosquitoes which feed outdoors. Am J Trop Med Hyg.

[11] Wang SJ, Lengeler C, Smith TA, Vounatsou P, Cisse G, Diallo DA (2005). Rapid urban malaria appraisal (RUMA) in sub-Saharan Africa. Malar J..

[12] Fillinger U, Kannady K, William G, Vanek MJ, Dongus S, Nyika D (2008). A tool box for operational mosquito larval control: preliminary results and early lessons from the Urban Malaria Control Programme in Dar es Salaam, Tanzania. Malar J..

[13] Chaki PP, Kannady K, Mtasiwa D, Tanner M, Mshinda H, Kelly AH (2014). Institutional evolution of a community-based programme for malaria control through larval source management in Dar es Salaam, United Republic of Tanzania: a case study. Malar J..

[14] Ogoma SB, Kannady K, Sikulu M, Chaki PP, Govella NJ, Mukabana WR (2009). Window screening, ceilings and closed eaves as sustainable ways to control malaria in Dar es Salaam, Tanzania. Malar J..

[15] Maheu-Giroux M, Castro MC (2013). Impact of community-based larviciding on the prevalence of malaria infection in Dar es Salaam, Tanzania. PLoS One.

[16] Maheu-Giroux M, Castro MC (2013). Do malaria vector control measures impact disease-related behaviour and knowledge? Evidence from a large-scale larviciding intervention in Tanzania. Malar J..

[17] National Bureau of Statistics. The 2012 population and housing census. Government of the United Republic of Tanzania; 2013.

[18] UN-Habitat. State of the World Cities. Harmonious Cities 2008/2009. Nairobi: United Nations Human Settlements Development Programme; 2008.

[19] UN-Habitat. State of the World’s Cities 2010/2011—Cities for all: bridging the urban divide. Nairobi: United Nations Human Settlements Development Programme; 2010.

[20] Lupala J. The spatial dimension of urbanisation in least industrialised countries: analysis of the spatial growth of Dar es Salaam City, Tanzania. Weihai, 2003.

[21] Bates I, Fenton C, Gruber J, Lalloo D, Medina Lara A, Squire SB (2004). Vulnerability to malaria, tuberculosis and HIV/AIDS infection and disease. Part 1: determinants operating at individual and household level. Lancet Infect Dis..

[22] Bates I, Fenton C, Gruber J, Lalloo D, Medina Lara A, Squire SB (2004). Vulnerability to malaria, tuberculosis and HIV/AIDS infection and disease. Determinants operating at environmental and institutional level. Lancet Infect Dis..

[23] Dongus S, Nyika D, Kannady K, Mtasiwa D, Mshinda H, Fillinger U (2007). Participatory mapping of target areas to enable routine comprehensive larviciding of malaria vector mosquitoes in Dar es Salaam, Tanzania. Int J Health Geogr..

[24] Dongus S, Mwakalinga V, Kannady K, Tanner M, Killeen GF. Participatory mapping as a component of operational malaria vector control in Tanzania. In: Maantay JA, McLafferty S, editors. Geospatial analysis of environmental health. 2011. p. 321–36.

[25] Wang SJ, Lengeler C, Mtasiwa D, Mshana T, Manane L, Maro G (2006). Rapid Urban Malaria Appraisal (RUMA) II: epidemiology of urban malaria in Dar es Salaam (Tanzania). Malar J..

[26] Mtasiwa D, Seigfreid G, Tanner M, Pichette P (2003). The Dar es Salaam city/region minimum package of health and related management activities: From managing diseases to managing health systems.

[27] Stephens C, Masamu ET, Kiama MG, Keto AJ, Kinenekejo M, Ichimori K (1995). Knowledge of mosquitoes in relation to public and domestic control activities in the cities of Dar es Salaam and Tanga. Bull World Health Organ.

[28] Bang YH, Mrope FM, Sabuni IB (1977). Changes in mosquito populations associated with urbanization in Tanzania. East Afr Med J.

[29] Bang YH, Sabuni IB, Tonn RJ (1975). Integrated control of urban mosquitoes in Dar es Salaam using community sanitation supplemented by larviciding. East Afr Med J.

[30] Castro MC, Yamagata Y, Mtasiwa D, Tanner M, Utzinger J, Keiser J (2004). Integrated urban malaria control: a case study in Dar es Salaam, Tanzania. Am J Trop Med Hyg.

[31] Chavasse DC (1996). The relationship between mosquito density and mosquito coil sales in Dar es Salaam. Trans Roy Soc Trop Med Hyg..

[32] Chavasse DC, Lines JD, Ichimori K, Majala AR, Minjas JN, Marijani J (1995). Mosquito control in Dar es Salaam. II. Impact of expanded polystyrene beads and pyriproxyfen treatment of breeding sites on *Culex quinquefasciatus* densities. Med Vet Entomol.

[33] Chavasse DC, Lines JD, Ichimori K, Marijani J (1995). Mosquito control in Dar es Salaam. I. Assessment of *Culex quinquefasciatus* breeding sites prior to intervention. Med Vet Entomol.

[34] Kilama WL. Malaria in Tanzania: past and present. In Proceedings of the 11th Annual Joint Scientific Conference with a Seminar on Malaria Control Research. Arusha: National Institute for Medical Research; 1994.

[35] Clyde DF (1967). Malaria in Tanzania.

[36] Kilama WL, Mwaluko GMP, Kilama WL, Mandara PM, Murru M, MacPherson CNL (1991). Malaria. Health and Disease in Tanzania.

[37] Harpam T, Few R (2002). The Dar es Salaam Urban Health Project, Tanzania: a multi-dimensional evaluation. J Public Health Med.

[38] Mukabana WR, Kannady K, Kiama GM, Ijumba J, Mathenge EM, Kiche I (2006). Ecologists can enable communities to implement malaria vector control in Africa. Malar J..

[39] Alba S, Hetzel MW, Goodman C, Dillip A, Liana J, Mshinda H (2010). Improvements in access to malaria treatment in Tanzania after switch to artemisinin combination therapy and the introduction of accredited drug dispensing outlets—a provider perspective. Malar J..

[40] Geissbuhler Y, Kannady K, Chaki PP, Emidi B, Govella NJ, Mayagaya V (2009). Microbial larvicide application by a large-scale, community-based program reduces malaria infection prevalence in urban Dar es Salaam, Tanzania. PLoS One.

[41] Cohen JL, Yadav P, Moucheraud C, Alphs S, Larson PS, Arkedis J (2013). Do price subsidies on artemisinin combination therapy for malaria increase household use? Evidence from a repeated cross-sectional study in remote regions of Tanzania. PLoS One.

[42] Yadav P, Cohen JL, Alphs S, Arkedis J, Larson PS, Massaga J (2012). Trends in availability and prices of subsidized ACT over the first year of the AMFm: evidence from remote regions of Tanzania. Malar J..

[43] Thomson R, Festo C, Johanes B, Kalolella A, Bruxvoort K, Nchimbi H (2014). Has Tanzania embraced the green leaf? Results from outlet and household surveys before and after implementation of the affordable medicines facility-malaria. PLoS One.

[44] Rutta E, Kibassa B, McKinnon B, Liana J, Mbwasi R, Mlaki W (2011). Increasing sccess to subsidized artemisinin-based combination therapy through accredited drug dispensing outlets in Tanzania. Health Res Policy Syst..

[45] Makani J, Matuja W, Liyombo E, Snow RW, Marsh K, Warrell DA (2003). Admission diagnosis of cerebral malaria in adults in an endemic area of Tanzania: implications and clinical description. QJM.

[46] Kahama-Maro J, D’Acremont V, Mtasiwa D, Genton B, Lengeler C (2011). Low quality of routine microscopy for malaria at different levels of the health system in Dar es Salaam. Malar J..

[47] Yukich J, D’Acremont V, Kahama J, Swai N, Lengeler C (2010). Cost savings with rapid diagnostic tests for malaria in low-transmission areas: evidence from Dar es Salaam Tanzania. Am J Trop Med Hyg..

[48] D’Acremont V, Kahama-Maro J, Swai N, Mtasiwa D, Genton B, Lengeler C (2011). Reduction of anti-malarial consumption after rapid diagnostic tests implementation in Dar es Salaam: a before-after and cluster randomized controlled study. Malar J..

[49] Bruxvoort K, Kalolella A, Nchimbi H, Festo C, Taylor M, Thomson R (2013). Getting antimalarials on target: impact of national roll-out of malaria rapid diagnostic tests on health facility treatment in three regions of Tanzania. Trop Med Int Health..

[50] Mubi M, Janson A, Warsame M, Martensson A, Kallander K, Petzold MG (2011). Malaria rapid testing by community health workers is effective and safe for targeting malaria treatment: randomised cross-over trial in Tanzania. PLoS One.

[51] Castro MC, Kanamori S, Kannady K, Mkude S, Killeen GF, Fillinger U (2010). The Importance of drains for larval development of lymphatic filariasis and malaria vectors in Dar es Salaam, Tanzania. PLoS Neglect Trop Dis..

[52] Castro MC, Tsuruta A, Kanamori S, Kannady K, Mkude S (2009). Community-based environmental management for malaria control: evidence from a small-scale intervention in Dar es Salaam, Tanzania. Malar J..

[53] Worrall E, Fillinger U (2011). Large-scale use of mosquito larval source management for malaria control in Africa: a cost analysis. Malar J..

[54] Chaki PP, Dongus S, Kannady K, Fillinger U, Kelly A, Killeen GF (2011). Community-owned resource persons for malaria vector control: enabling factors and challenges in an operational programme in Dar es Salaam, Tanzania. Human Res Health..

[55] Chaki PP, Govella NJ, Shoo B, Hemed A, Tanner M, Fillinger U (2009). Achieving high coverage of larval-stage mosquito surveillance: challenges for a community-based mosquito control programme in urban Dar es Salaam, Tanzania. Malar J..

[56] Chaki PP, Mlacha Y, Msellemu D, Muhili A, Malishee AD, Mtema ZJ (2012). An affordable, quality-assured, community-based system for high resolution entomological surveillance of vector mosquitoes that reflects human malaria infection risk patterns. Malar J..

[57] Kelly AH, Lezaun J (2014). Urban mosquitoes, situational publics, and the pursuit of interspecies separation in Dar es Salaam. Amer Ethnol..

[58] PMI. FY09 Malaria Operational Plan (MOP) Tanzania. Washington; 2008. p. 65.

[59] Magesa SM, Lengeler C, deSavigny D, Miller JE (2005). Creating an “enabling environment” for taking insecticide treated nets to national scale: the Tanzanian experience. Malar J..

[60] Bonner K, Mwita A, McElroy PD, Omari S, Mzava A, Lengeler C (2011). Design, implementation and evaluation of a national campaign to distribute nine million free LLINs to children under five years of age in Tanzania. Malar J..

[61] Renggli S, Mandike R, Kramer K, Patrick F, Brown NJ, McElroy PD (2013). Design, implementation and evaluation of a national campaign to deliver 18 million free long-lasting insecticidal nets to uncovered sleeping spaces in Tanzania. Malar J..

[62] Zhou Y, Lobo NF, Wolkon A, Gimnig JE, Malishee A, Stevenson J (2014). PGMS: a case study of collecting PDA-based geo-tagged malaria-related survey data. Am J Trop Med Hyg.

[63] Corran PH, Cook J, Lynch C, Leendertse H, Manjurano A, Griffin J (2008). Dried blood spots as a source of anti-malarial antibodies for epidemiological studies. Malar J..

[64] Ishengoma DS, Lwitiho S, Madebe RA, Nyagonde N, Persson O, Vestergaard LS (2011). Using rapid diagnostic tests as source of malaria parasite DNA for molecular analyses in the era of declining malaria prevalence. Malar J..

[65] Govella NJ, Moore JD, Killeen GF (2010). An exposure free tool for monitoring adult malaria mosquito populations. Am J Trop Med Hyg.

[66] Govella NJ, Chaki P, Mpangile JM, Killeen GF (2011). Monitoring mosquitoes in urban Dar es Salaam: evaluation of resting boxes, window exit traps, CDC light traps, Ifakara tent traps and human landing catches. Parasit Vectors..

[67] Govella NJ, Chaki PP, Geissbuhler Y, Kannady K, Okumu F, Charlwood JD (2009). A new tent trap for sampling exophagic and endophagic members of the *Anopheles gambiae* complex. Malar J..

[68] Majambere S, Massue DJ, Mlacha Y, Govella NJ, Magesa SM, Killeen GF (2013). Advantages and limitations of commercially available electrocuting grids for studying mosquito behaviour. Parasit Vectors..

[69] Govella NJ, Ogoma SB, Paliga J, Chaki PP, Killeen G (2015). Impregnating hessian strips with the volatile pyrethroid transfluthrin prevents outdoor exposure to vectors of malaria and lymphatic filariasis in urban Dar es Salaam, Tanzania. Parasit Vectors..

[70] Burkot TR, Williams JL, Schneider I (1984). Identification of *Plasmodium falciparum*-infected mosquitoes by a double antibody enzyme-linked immunosorbent assay. Am J Trop Med Hyg.

[71] Durnez L, Van Bortel W, Denis L, Roelants P, Veracx A, Trung HD (2011). False positive circumsporozoite protein ELISA: a challenge for the estimation of the entomological inoculation rate of malaria and for vector incrimination. Malar J..

[72] Scott JA, Brogdon WG, Collins FH (1993). Identification of single specimens of the *Anopheles gambiae* complex by the polymerase chain reaction. Am J Trop Med Hyg.

[73] Bradley J, Lines J, Fuseini G, Schwabe C, Monti F, Slotman MA (2015). Outdoor biting by *Anopheles* mosquitoes on Bioko Island does not currently impact on malaria control. Malar J..

[74] Seyoum A, Sikaala CH, Chanda J, Chinula D, Ntamatungiro AJ, Hawela M (2012). Most exposure to *Anopheles funestus* and *Anopheles quadriannulatus* in Luangwa valley, South-east Zambia occurs indoors, even for users of insecticidal nets. Parasit Vectors..

[75] Huho BJ, Briët O, Seyoum A, Sikaala CH, Bayoh N, Gimnig JE (2013). Consistently high estimates for the proportion of human exposure to malaria vector populations occurring indoors in rural Africa. Int J Epidemiol.

[76] Killeen GF, Kihonda J, Lyimo E, Okech FR, Kotas ME, Mathenge E (2006). Quantifying behavioural interactions between humans and mosquitoes: evaluating the protective efficacy of insecticidal nets against malaria transmission in rural Tanzania. BMC Infect Dis.

[77] Govella NJ, Chaki PP, Killeen GF (2013). Entomological surveillance of behavioural resilience and resistance in residual malaria vector populations. Malar J..

[78] Kiswewski AE, Mellinger A, Spielman A, Malaney P, Sachs SE, Sachs J (2004). A global index representing the stability of malaria transmission. Am J Trop Med Hyg.

[79] Abba KDJ, Olliaro PL, Naing CM, Jackson SM, Takwoingi Y, Donegan S (2011). Rapid diagnostic tests for diagnosing uncomplicated *Plasmodium falciparum* malaria in endemic countries. Cochrane Database Syst Rev.

[80] Wilson AL, Boelaert M, Kleinschmidt I, Pinder M, Scott TW, Tusting LS (2015). Evidence-based vector control? Improving the quality of vector control trials. Trend Parasitol.

[81] Habicht JP, Victora CG, Vaughan JP (1999). Evaluation designs for adequacy, plausibility and probability of public health programme performance and impact. Int J Epidemiol.

[82] Govella NJ, Ferguson HM (2012). Why use of interventions targetting outdoor biting mosquitoes will be necessary to achieve malaria elimination. Front Physiol..

[83] Moiroux N, Damien GB, Egrot M, Djenontin A, Chandre F, Corbel V (2014). Human exposure to early morning *Anopheles funestus* biting behavior and personal protection provided by long-lasting insecticidal nets. PLoS One.

[84] Sougoufara S, Diedhiou SM, Doucoure S, Diagne N, Sembene PM, Harry M (2014). Biting by *Anopheles funestus* in broad daylight after use of long-lasting insecticidal nets: a new challenge to malaria elimination. Malar J..

[85] Deressa W, Yihdego YY, Kebede Z, Batisso E, Tekalegne A, Dagne GA (2014). Effect of combining mosquito repellent and insecticide treated net on malaria prevalence in Southern Ethiopia: a cluster-randomised trial. Parasit Vectors..

[86] Pates HV, Line JD, Keto AJ, Miller JE (2002). Personal protection against mosquitoes in Dar es Salaam, Tanzania, by using a kerosene oil lamp to vaporize transfluthrin. Med Vet Entomol.

[87] Achee NL, Bangs MJ, Farlow R, Killeen GF, Lindsay S, Logan JG (2012). Spatial repellents: from discovery and development to evidence-based validation. Malar J..

[88] Macintyre K, Sosler S, Letipila F, Lochigan M, Hassig S, Omar SA (2003). A new tool for malaria prevention? Results of a trial of permethrin-impregnated bedsheets (shukas) in an area of unstable transmission. Int J Epidemiol.

[89] Kimani EW, Vulule JM, Kuria IW, Mugisha F (2006). Use of insecticide-treated clothes for personal protection against malaria: a community trial. Malar J..

[90] Kitau J, Oxborough R, Kaye A, Chen-Hussey V, Isaacs E, Matowo J (2014). Laboratory and experimental hut evaluation of a long-lasting insecticide treated blanket for protection against mosquitoes. Parasit Vectors..

[91] Chen I, Clarke SE, Gosling R, Hamainza B, Killeen G, Magill A (2016). “Asymptomatic” malaria: a chronic and debilitating infection that should be treated. PLoS Med..

[92] Beier JC, Killeen GF, Githure J (1999). Short report: entomologic inoculation rates and *Plasmodium falciparum* malaria prevalence in Africa. Am J Trop Med Hyg.

[93] Smith DL, Dushoff J, Snow RW, Hay SI (2005). The entomological inoculation rate and *Plasmodium falciparum* infection in African children. Nature.

[94] Smith DL, McKenzie FE, Snow RW, Hay SI (2007). Revisiting the basic reproductive number for malaria and its implications for malaria control. PLoS Biol.

[95] Krogstad DJ, Koita OA, Diallo M, Gerone JL, Poudiougou B, Diakite M (2015). Molecular incidence and clearance of *Plasmodium falciparum* infection. Malar J..

[96] Escalante AA, Ferreira MU, Vinetz JM, Volkman SK, Cui L, Gamboa D (2015). Malaria molecular epidemiology: lessons from the International Centers of Excellence for Malaria Research Network. Am J Trop Med Hyg.

[97] Cox J, Hay SI, Abeku TA, Checchi F, Snow RW (2007). The uncertain burden of *Plasmodium falciparum* epidemics in Africa. Trends Parasitol.

[98] Smith DL, Cohen JM, Chiyaka C, Johnston G, Gething PW, Gosling R (2013). A sticky situation: the unexpected stability of malaria elimination. Philos Trans R Soc Lond B Biol Sci.

[99] Bousema T, Okell L, Felger I, Drakeley C (2014). Asymptomatic malaria infections: detectability, transmissibility and public health relevance. Nat Rev Microbiol.

[100] Bretscher MT, Maire N, Chitnis N, Felger I, Owusu-Agyei S, Smith T (2011). The distribution of *Plasmodium falciparum* infection durations. Epidemics..

[101] Sama W, Dietz K, Smith T (2006). Distribution of survival times of deliberate *Plasmodium falciparum* infections in tertiary syphilis patients. Trans R Soc Trop Med Hyg.

[102] Sama W, Killeen GF, Smith T (2004). Estimating the duration of *Plasmodium falciparum* infection from malaria eradication trials. Am J Trop Med Hyg.

[103] Smith DL, Hay SI (2009). Endemicity response timelines for *Plasmodium falciparum* elimination. Malar J..

[104] Ashley EA, White NJ (2014). The duration of *Plasmodium falciparum* infections. Malar J..

[105] Maheu-Giroux M, Castro MC (2014). Cost-effectiveness of larviciding for urban malaria control in Tanzania. Malar J..

[106] Killeen GF (2013). A second chance to tackle African malaria vector mosquitoes that avoid houses and don’t take drugs. Am J Trop Med Hyg.

[107] Killeen GF (2014). Characterizing, controlling and eliminating residual malaria transmission. Malar J..

[108] Killeen GF, Seyoum A, Gimnig JE, Stevenson JC, Drakeley CJ, Chitnis N (2014). Made-to-measure malaria vector control strategies: rational design based on insecticide properties and coverage of blood resources for mosquitoes. Malar J..

[109] Killeen GF, Seyoum A, Sikaala CH, Zomboko AS, Gimnig JE, Govella NJ (2013). Eliminating malaria vectors. Parasit Vectors..

[110] Kabula B, Tungu P, Malima R, Rowland M, Minja J, Wililo R (2014). Distribution and spread of pyrethroid and DDT resistance among the *Anopheles gambiae* complex in Tanzania. Med Vet Entomol.

